# The usefulness of SwiftScan technology for bone scintigraphy using a novel anthropomorphic phantom

**DOI:** 10.1038/s41598-021-82082-x

**Published:** 2021-01-29

**Authors:** Takayuki Shibutani, Masahisa Onoguchi, Yuka Naoi, Hiroto Yoneyama, Takahiro Konishi, Ringo Tatami, Kenichi Nakajima

**Affiliations:** 1 Department of Quantum Medical Technology, Institute of Medical, Pharmaceutical and Health Sciences, Kanazawa University, Kanazawa, Japan; 2 Clinical Imaging Center for Healthcare, Nippon Medical School, Tokyo, Japan; 3 Department of Radiological Technology, Kanazawa University Hospital, Kanazawa, Japan; 4 Department of Radiological Technology, Ishikawa Prefectural Central Hospital, Kanazawa, Japan; 5 Department of Functional Imaging and Artificial Intelligence, Kanazawa University, Kanazawa, Japan

**Keywords:** Medical research, Molecular medicine, Materials science

## Abstract

The aim of this study was to demonstrate the usefulness of SwiftScan with a low-energy high-resolution and sensitivity (LEHRS) collimator for bone scintigraphy using a novel bone phantom simulating the human body. SwiftScan planar image of lateral view was acquired in clinical condition; thereafter, each planar image of different blend ratio (0–80%) of Crality 2D processing were created. SwiftScan planar images with reduced acquisition time by 25–75% were created by Poisson’s resampling processing. SwiftScan single photon emission computed tomography (SPECT) was acquired with step-and-shoot and continuous mode, and SPECT images were reconstructed using a three-dimensional ordered subset expectation maximization incorporating attenuation, scatter and spatial resolution corrections. SwiftScan planar image showed a high contrast to noise ratio (CNR) and low percent of the coefficient of variance (%CV) compared with conventional planar image. The CNR of the tumor parts in SwiftScan SPECT was higher than that of the conventional SPECT image of step and shoot acquisition, while the %CV showed the lowest value in all systems. In conclusion, SwiftScan planar and SPECT images were able to reduce the image noise compared with planar and SPECT image with a low-energy high-resolution collimator, so that SwiftScan planar and SPECT images could be obtained a high CNR. Furthermore, the SwiftScan planar image was able to reduce the acquisition time by 25% when the blend ratio of Clarity 2D processing set to more than 40%.

## Introduction

Bone scintigraphy is a highly sensitive diagnostic nuclear medicine imaging technique using a radiotracer to evaluate the distribution of active bone formation in the skeleton related to malignant and benign diseases^1–3^. Planar whole-body bone images in anterior and posterior views are always acquired as a screening examination to detect bone diseases such as metastasis, inflammation and necrosis. In addition, bone single photon emission computed tomography (SPECT) or SPECT/computed tomography (SPECT/CT) image has been added to improve diagnostic accuracy^4^. In particular, multimodality bone SPECT/CT offers the unique opportunity to correlate planar image findings with anatomical images and introduces novel algorithms to further enhance SPECT image quality by CT-based attenuation and scatter corrections^5^. This results in an improved correlation of areas with physiological variants or abnormal tracer accumulation with anatomical landmarks. While the total examination time is longer by a variety of bone scans, the increase of examination time leads to the increase of patient motion and burden. Therefore, the acquisition time of planar and SPECT scans must be reduced while maintaining image quality.

A novel low-energy high-resolution and sensitivity (LEHRS) collimator was developed by General Electric Healthcare (GE Healthcare, Milwaukee, WI, USA). The designs of low-energy high-resolution (LEHR) and LEHRS collimator were 1.5 and 1.43 mm for a hole diameter, 0.2 and 0.13 mm septal thickness, and 35 and 32 mm for hole length, respectively. Furthermore, the spatial resolution and system sensitivity were 7.4 mm and 72 cps/MBq for LEHR collimator, and 7.4 mm and 92 cps/MBq for LEHRS collimator, respectively. The LEHRS collimator has a high sensitivity to realize the thin septum thickness and short hole length. The SwiftScan system introduced as a new acquisition and image processing using the LEHRS collimator, and it was proposed to achieve low-dose or short-term acquisition. SwiftScan system is classified into SwiftScan planar and SPECT. SwiftScan planar image was Clarity 2D processing incorporating three procedures of noise reduction, contrast enhancement and blending against planar or whole-body images^6–9^. The blend ratio of Clarity 2D processing indicated the percentage of the original and the processed images after noise reduction and contrast enhance as shown in the following equation:

$${\text{Pixel}}_{{\text{c}}} = \left( {{1} - {\text{W}}_{{\text{p}}} } \right) \cdot {\text{Pixel}}_{{\text{o}}} + {\text{W}}_{{\text{p}}} \cdot {\text{Pixel}}_{{\text{p}}}$$

where, pixel_c_, pixel_o_ and pixel_p_ are pixel value of Clarity 2D image, the original image and the processed image after noise reduction and contrast enhancement, W_p_ is the weight (0–100%) of the processed image and considered as the Clarity 2D blending weight.

The step-and-shoot mode of conventional SPECT does not acquire while moving between detectors, which results in dead time. Meanwhile, SwiftScan SPECT can be acquired data even while moving between detectors, which is different from conventional SPECT with step-and-shoot mode. The projection data obtained by the detector-to-detector movement is divided into half data. If the step angle is acquired at 6°, the projection data is divided into the data from 0° to 3° and the data from 4° to 6°. The data from 0° to 3° is then added to the projection view before detector movement, and the data from 4° to 6° is added to the projection view after detector movement. As a result, SwiftScan SPECT can increase the counts by acquiring during detector movement in addition to the conventional step-and-shoot mode.

Although clinical and phantom studies using the SwiftScan system have already been reported, physical evaluation has been not fully evaluated^10^. In particular, the difference of blend ratio is important in determining the image quality. Furthermore, we used a novel anthropomorphic phantom that can simulate the attenuation and scatter of supine bone^11,12^. The aim of this study was to demonstrate the usefulness of SwiftScan planar and SPECT for bone scintigraphy using a novel anthropomorphic phantom.

## Material and methods

### Phantom design

**Figure 1:**
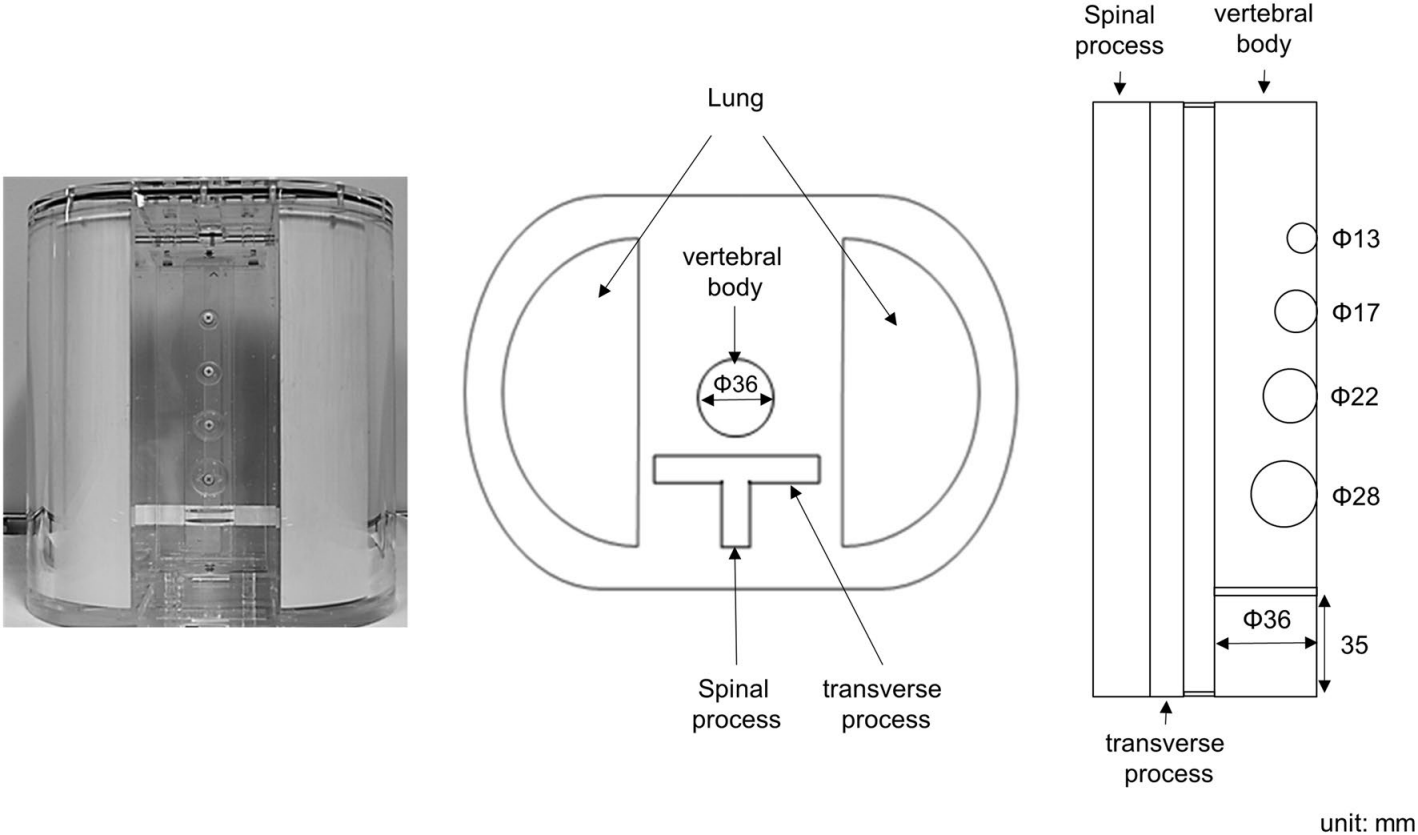
An overview of Sim^2^ Bone phantom. The left image shows the appearance of the phantom. The middle and right diagrams show the schemes of transverse and lateral views.

Phantom was used as a Sim^2^ bone phantom (Kyoto Kagaku, Co., Ltd., Japan), which simulated the thorax portion including the spine, mediastinum and lung (Fig. 1). The thorax phantom was constructed with an elliptical shape with a major axis of 310 mm, a minor axis of 210 mm, and a height of 320 mm, in which the lungs and supine portions were inserted. The vertebral body was constructed in a cylinder shape with a diameter of 36 mm and a height of 207 mm, and the spinous and transverse processes were T-shaped containers with a major axis of 90 mm, a minor axis of 40 mm, and a height of 250 mm. In addition, there are different five tumor regions (the diameter of the sphere: 13, 17, 22 and 28 mm), and the whole vertebral body (Reference: diameter of 36 mm and length of 35 mm) in the vertebral body. The lungs were semicircular columns with 170 mm in diameter and height of 300 mm.

We prepared a dipotassium hydrogen phosphate (K_2_HPO_4_) solution of 1.5 g/mL to simulate the cortical bone^13^. Tumor and normal bone parts were filled with a mixed solution of ^99m^Tc and K_2_HPO_4_ of 300 and 50 kBq/mL, respectively. The body part was also filled with a ^99m^Tc solution of 8 kBq/mL as a background (BG). The radioactive concentration ratios of tumor, normal and BG portions were 6:1:0.16^14^.

### Acquisition and image reconstruction parameters

We used a dual-head Discovery NM/CT670 Q.suite Pro (GE Healthcare, Milwaukee, USA) SPECT/CT scanner equipped with LEHR and LEHRS collimators. The planar scan of lateral view was acquired with 140.5 keV ± 7.5% of photo-peak window width, 256 × 256 matrices, 2.2 mm of pixel size, and 3 min of total scan time. Clarity 2D processing was applied to five planar images (blend ratio = 0%, 20%, 40%, 60% and 80%) with LEHRS to evaluate SwiftScan planar image. In addition, we created three planar images simulating one-fourth time (25% acquisition), half time (50% acquisition), and three-fourth time (75% acquisition) acquisitions as the clinical condition time (100% acquisition) using a technique called ‘Poisson resampling’^15–17^. The photo-peak and scatter windows of SPECT scan were set to 140.5 keV ± 10% and 120 keV ± 5%, and acquired with 128 × 128 matrices, 4.42 mm of pixel size, the ellipse orbit of 360° in 6° increments. We drew the rectangle region of interest (ROI) on the posterior view of projection data, and the average normal thoracic spine bone counts were regulated to 15 counts/pixel with reference to the clinical image. Low-dose CT scan images were then acquired using adaptive dose modulation with 3.75-mm slice thickness at 120 kVp and 20 mAs.

SPECT images with LEHR and LEHRS collimators were reconstructed by three-dimensional ordered subset expectation maximization (3D-OSEM) methods incorporating Evolution for bone algorithm^18,19^. The number of subsets and iterations were 10 and 5, respectively. A Gaussian filter with 8.84 mm of full width at half maximum (FWHM) was used as a post-filter. A scatter correction was conducted using the dual-energy window method^20^. In addition, attenuation correction was applied using a patient-dedicated low-dose CT-derived μ-map.

### Image assessment

**Figure 2:**
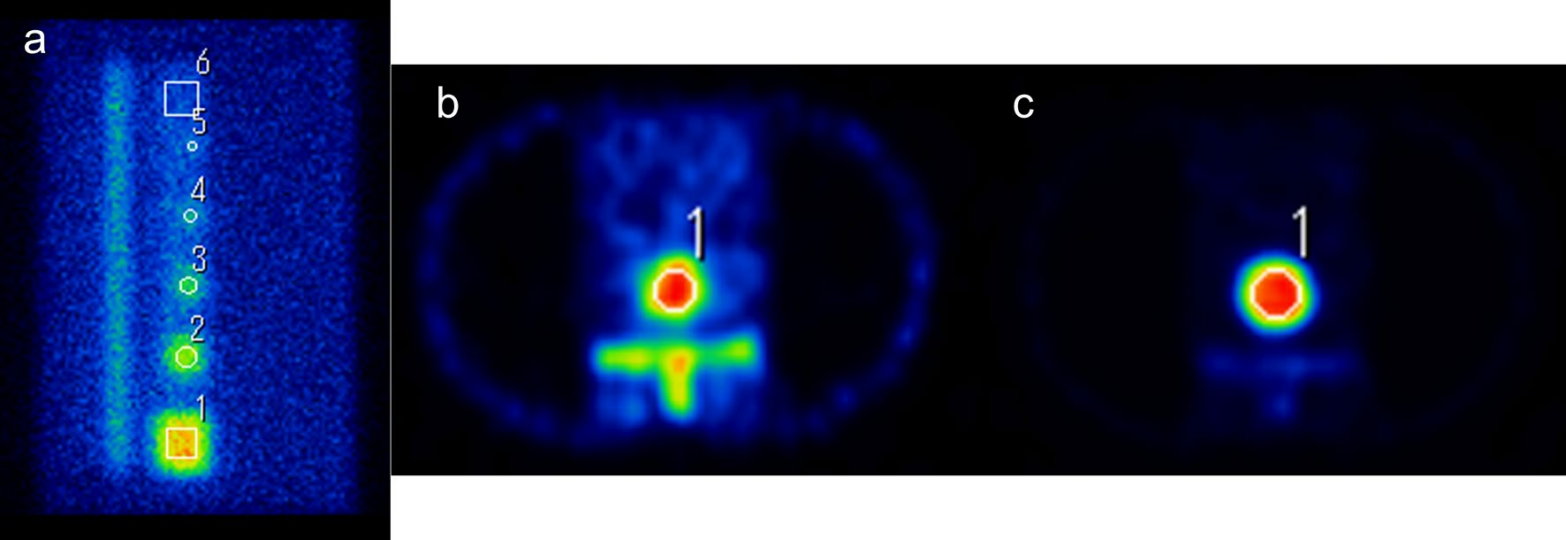
Planar and SPECT images calculating contrast to noise ratio (CNR) and the percent of the coefficient of variance (%CV). (**a**) Lateral view image with SwiftScan planar, transverse image of (**b**) tumor bone and (**c**) normal bone. Circle or rectangle ROI was set to each tumor and normal bones in planar and SPECT images using Prominence processor version 3.1 software (http://nm.jsrt.or.jp/blog.html) provided by Japanese Society of Radiological Technology’s subgroup Nuclear Medicine Section.

The ROI was drawn on tumor and normal bone parts of the planar and transverse images using Prominence processor version 3.1 software (http://nm.jsrt.or.jp/blog.html) provided by Japanese Society of Radiological Technology’s subgroup Nuclear Medicine Section (Fig. 2), and contrast ratio and contrast to noise ratio (CNR) were calculated using the following Eqs. 1 and 2:

1 $${\text{Contrast}}\,{\text{ratio}} = \frac{{\text{B}}}{{\text{A}}}$$

2 $${\text{CNR}} = \frac{{{\text{B}} - {\text{A}}}}{{{\text{SD}}_{{\text{A}}} }}$$

where A and B are the average counts in ROI on normal and tumor bone parts, and SD_A_ is the standard deviation (SD) of the average counts (A) in ROI on the normal bone part. The percent of the coefficient of variation (%CV) was calculated from the average counts (B) and SD_B_ of each tumor bone part.

In addition, two expert technologists who had experience in nuclear medicine with more than 15 years performed the visual assessment of image uniformity and detectability of tumor to determine optimal acquisition time of planar image with and without Clarity 2D processing. Image uniformity was scored with a five-point scale (1, bad; 2, poor; 3, even; 4, good; 5, excellent), and an average score of optimal image uniformity was defined as 4 or more points. Detectability was evaluated with the visible minimum diameter in different five tumor regions, and the final judgment of visible minimum diameter was performed by the consensus of two expert technologists. Since the spatial resolution of the current SPECT system is around 17 mm for bone SPECT, optimal image quality was defined that the tumor region of diameter < 17 mm can be clearly visualized, which is recommended by the Japanese Society of Nuclear Medicine Technology^14^.

### Clinical cases for SwiftScan planar and SPECT images

We present the case of a 76-year-old man with a documented history of hemosputum and carcinoma of the lung who was referred for whole-body bone scintigraphy for staging. The whole-body (WB) scan of scan speed 15 cm/min using LEHRS collimator was performed 3 h after intravenous injection of ^99m^Tc- hydroxymethylene diphosphonate (740 MBq) into the left antecubital vein. We performed the Clarity 2D processing using blend ratios of 20%, 40%, 60% and 80% on WB images as well as phantom study. Subsequently, the SPECT-CT scan using the LEHRS collimator was carried out under the same acquisition and image processing parameters of the phantom with and without SwiftScan. We set the ROIs on abnormal and BG regions, and CNR was calculated. The study protocol was approved by the institutional ethics committee of Kanazawa University. All data acquisition methods used in this study were in accordance with international, national, and institutional guidelines. Prior to beginning the protocol, all participants gave informed, written consent.

### Statistical analysis

Statistical analysis was performed using a statistical package for social science (SPSS) version 24 for Windows (SPSS Inc., Chicago, IL, USA) software program. The contrast ratio, CNR and %CV of planar images with and without Clarity 2D processing with an increase in a blend ratio of Clarity 2D processing were performed, and trend analysis using Jonckheere-Terpstra test. All statistical tests were two-tailed, and a *p* value of less than 0.05 was considered significantly different.

## Results

**Figure 3:**
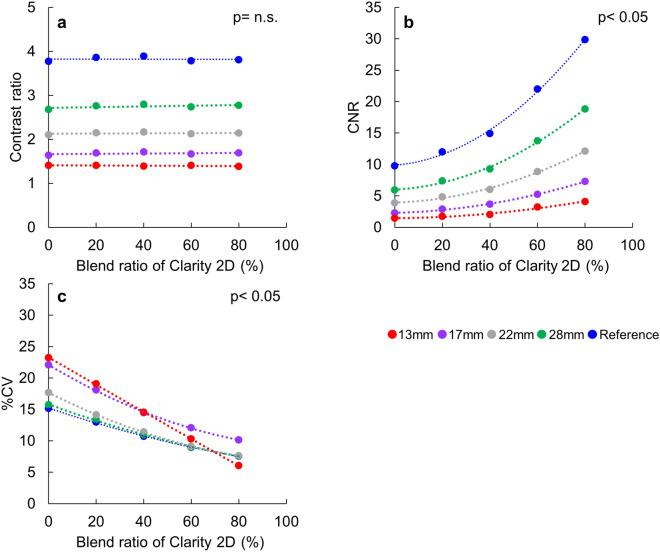
Quantitative evaluation of different blend ratios for Clarity 2D processing in the SwiftScan planar image. (**a**) Contrast ratio, (**b**) Contrast to noise ratio (CNR), and (**c**) the percent of the coefficient of variance (% CV).

Figure 3 showed the contrast ratio, CNR and %CV of phantom study. The contrast ratio of 13, 17, 22, 28 mm and reference tumor parts for planar images was 1.2, 1.7, 2.0, 2.8 and 4.0 with LEHR collimator and 1.4, 1.6, 2.1, 2.7 and 3.8 for LEHRS without Clarity 2D processing, respectively. The contrast ratio of 13, 17, 22, 28 mm and reference tumor parts for SwiftScan planar image with Clarity 2D processing was 1.40 ± 0.01, 1.68 ± 0.03, 2.14 ± 0.03, 2.75 ± 0.04 and 3.82 ± 0.05, respectively. The LEHR, LEHRS and SwiftScan planar images showed a similar contrast ratio among different tumor regions. Furthermore, the contrast ratio for different blend ratios of Clarity 2D processing had no trend by Clarity 2D blend ratio for all tumor regions (Fig. 3a: p = n.s.). The CNR of 13, 17, 22, 28 mm and reference tumor parts for planar images was 0.6, 2.1, 3.0, 5.2 and 9.0 with LEHR collimator and 1.4, 2.3, 3.9, 5.9 and 9.8 for LEHRS collimator without Clarity 2D processing, respectively. The CNR of 13, 17, 22, 28 mm and reference tumor parts for SwiftScan planar image with Clarity 2D processing was 1.4 – 4.1, 2.3 – 7.3, 3.9 – 12.1, 5.9 – 18.8 and 9.8 – 29.9, respectively (Fig. 3b). Although the CNR of LEHR and LEHRS collimators did not differ the CNR of the SwiftScan planar image significantly showed a significantly higher value with an increase in a blend ratio of Clarity 2D processing (*p* < 0.05). The %CV of 13, 17, 22, 28 mm and reference tumor parts for planar images was 33.9%, 24.1%, 26.1%, 19.5% and 15.5% with LEHR collimator and 23.2%, 22.1%, 17.7%, 15.7% and 15.2% for LEHRS collimator without Clarity 2D processing, respectively. The %CV of 13, 17, 22, 28 mm and reference tumor parts for SwiftScan planar image with Clarity 2D processing was 6.0 – 23.2%, 10.1 – 22.1%, 7.6 – 17.7%, 7.6 – 15.7% and 7.5 – 15.2%, respectively (Fig. 3c). The %CV of the LEHRS collimator was lower than that of the LEHR collimator. Furthermore, the %CV of the SwiftScan planar image showed a significantly lower value with an increase in a blend ratio of Clarity 2D processing (*p* < 0.05).

**Figure 4:**
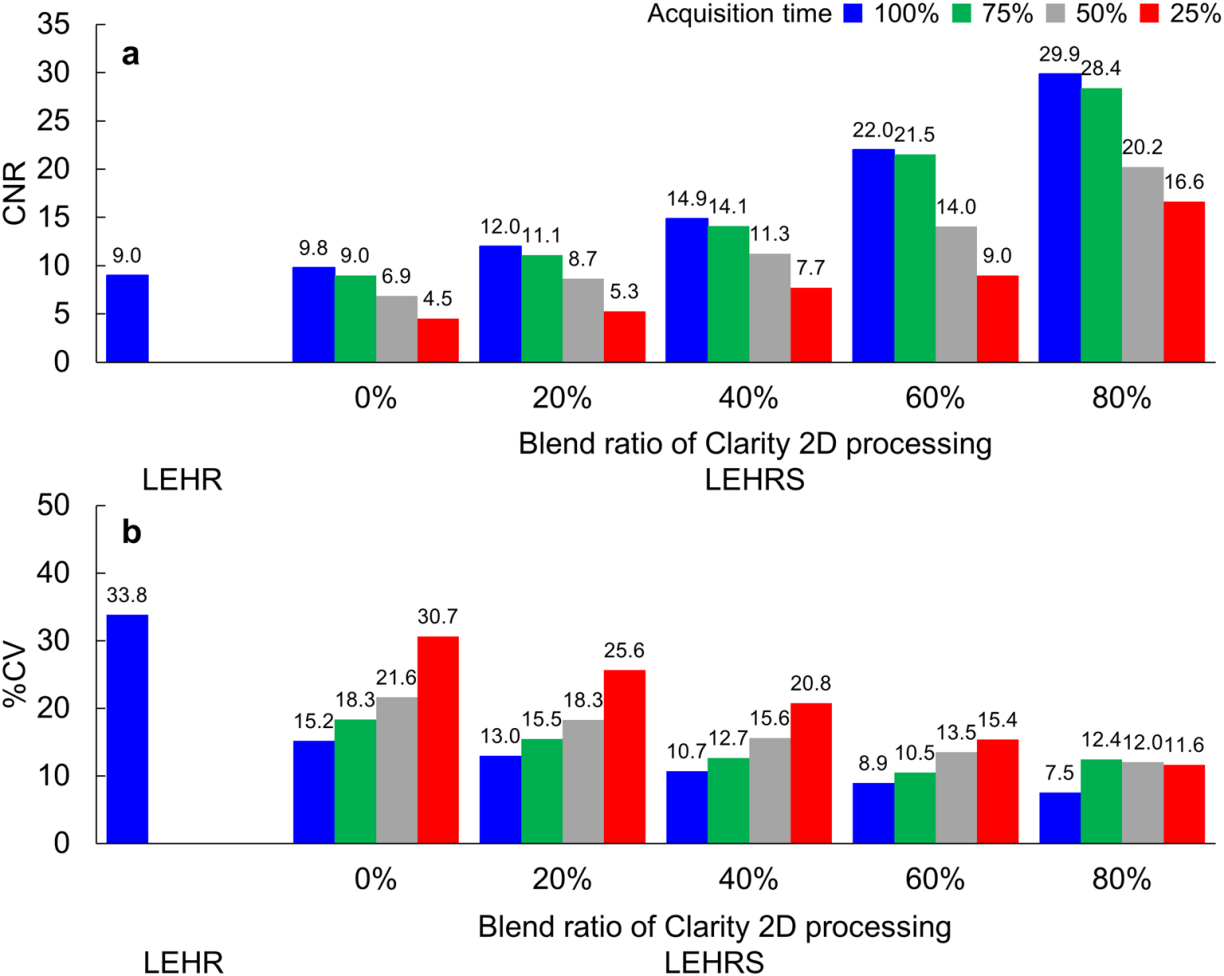
Quantitative evaluation of tumor bone with a reference part for different acquisition times in the SwiftScan planar image. (**a**) Contrat to noise ratio (CNR), (**b**) the percent of the coefficient of variance (% CV). *LEHR* low-energy high-resolution, *LEHRS* low-energy high-resolution and sensitivity.

**Figure 5:**
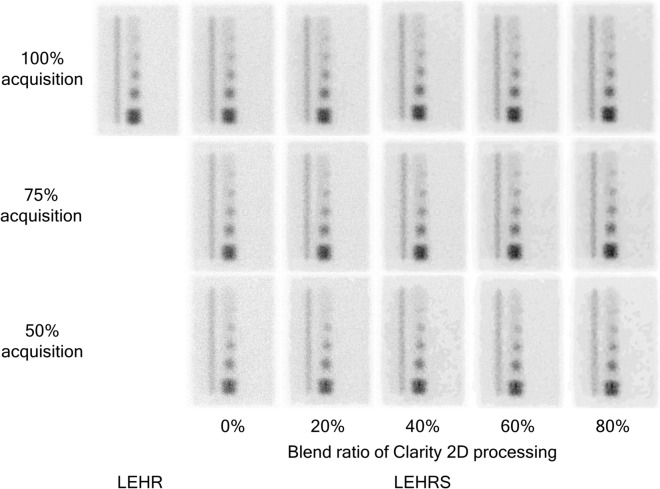
The phantom image of SwiftScan planar processing. Upper, middle and lower rows show the 100%, 75% and 50% acquisition time, respectively. The 100% acquisition corresponds to the clinical condition. *LEHR* low-energy high-resolution, *LEHRS* low-energy high-resolution and sensitivity.

The CNR of reference tumor parts for 100%, 75%, 50% and 25% acquisitions was 9.8, 9.0, 6.9 and 4.5 without Clarity 2D processing, and those by blend ratio of Clarity 2D processing were 12.0, 11.1, 8.7 and 5.3 for 20%, 14.9, 14.1, 11.3 and 7.7 for 40%, 22.0, 21.5, 14.0 and 9.0 for 60%, and 29.9, 28.4, 20.2 and 16.6 for 80%, respectively (Fig. 4a). The CNR with and without Clarity 2D processing was lower with a short acquisition. When the acquisition time was 50% and 75%, the SwiftScan planar image of the blend ratio more than 40% showed higher CNR in comparison to conventional planar images with LEHR and LEHRS collimators. The %CV of reference tumor parts for 100%, 75%, 50% and 25% acquisitions was 15.2%, 18.3%, 21.6% and 30.7% without Clarity 2D processing, and those by blend ratio of Clarity 2D processing were 13.0%, 15.5%, 18.3% and 25.6% for 20%, 10.7%, 12.7%, 15.6% and 20.8% for 40%, 8.9%, 10.5%, 13.5% and 15.4% for 60%, and 7.5%, 12.4%, 12.0% and 11.6% for 80%, respectively (Fig. 4b). The %CV with and without Clarity 2D processing was lower with a short acquisition. When the acquisition time was 50%, the SwiftScan planar image for the blend ratio of 40% or more showed a lower %CV in comparison to conventional planar images with LEHR and LEHRS collimators. In addition, when the blend ratio of Clarity 2D processing was more than 60%, the SwiftScan planar image of 25% acquisition showed a low %CV. Figure 5 shows the planar image with the lateral view. When the planar image was 100% and 75% acquisitions, an average visual score of uniformity showed more than 4.0 at the blend ratio of more than 40% (Table 1). The visible minimum diameter of the tumor region for 100%, 75% and 50% acquisition time was less than 17 mm at the blend ratio of more than 40%.

**Table 1:** Image uniformity score and visible minimum diameter of tumor region for visual assessment.

Blend ratio of Clarity 2D (%)	Visual score (uniformity)					Visible minimum diameter (mm)				
	0	20	40	60	80	0	20	40	60	80
100% acquisition	2.5	3.0	4.0	4.0	5.0	22	22	17	13	13
75% acquisition	1.5	2.5	4.0	5.0	5.0	22	17	17	17	13
50% acquisition	1.5	1.5	3.0	2.5	3.5	22	22	17	17	17
25% acquisition	1.0	1.0	1.0	2	3.5	22	22	22	22	17

The visual score of uniformity shows the average value by two expert technologists. The visible minimum diameter was determined by the consensus of two expert technologists.

The CNR of 13, 17, 22, 28 mm and reference tumor parts for transverse images of SPECT was 5.1, 12.8, 17.9, 18.7 and 22.9 for LEHR collimator, 7.1, 14.0, 19.2, 20.1 and 27.7 for LEHRS collimator, and 7.1, 14.3, 21.6, 22.3 and 30.1 for SwiftScan SPECT with LEHRS collimator, respectively. The %CV of reference tumor parts of LEHR, LEHRS and SwiftScan SPECT was 22.9%, 16.3% and 15.6%, respectively. SwiftScan SPECT image showed the highest CNR and lowest %CV in all acquisitions.

**Figure 6:**
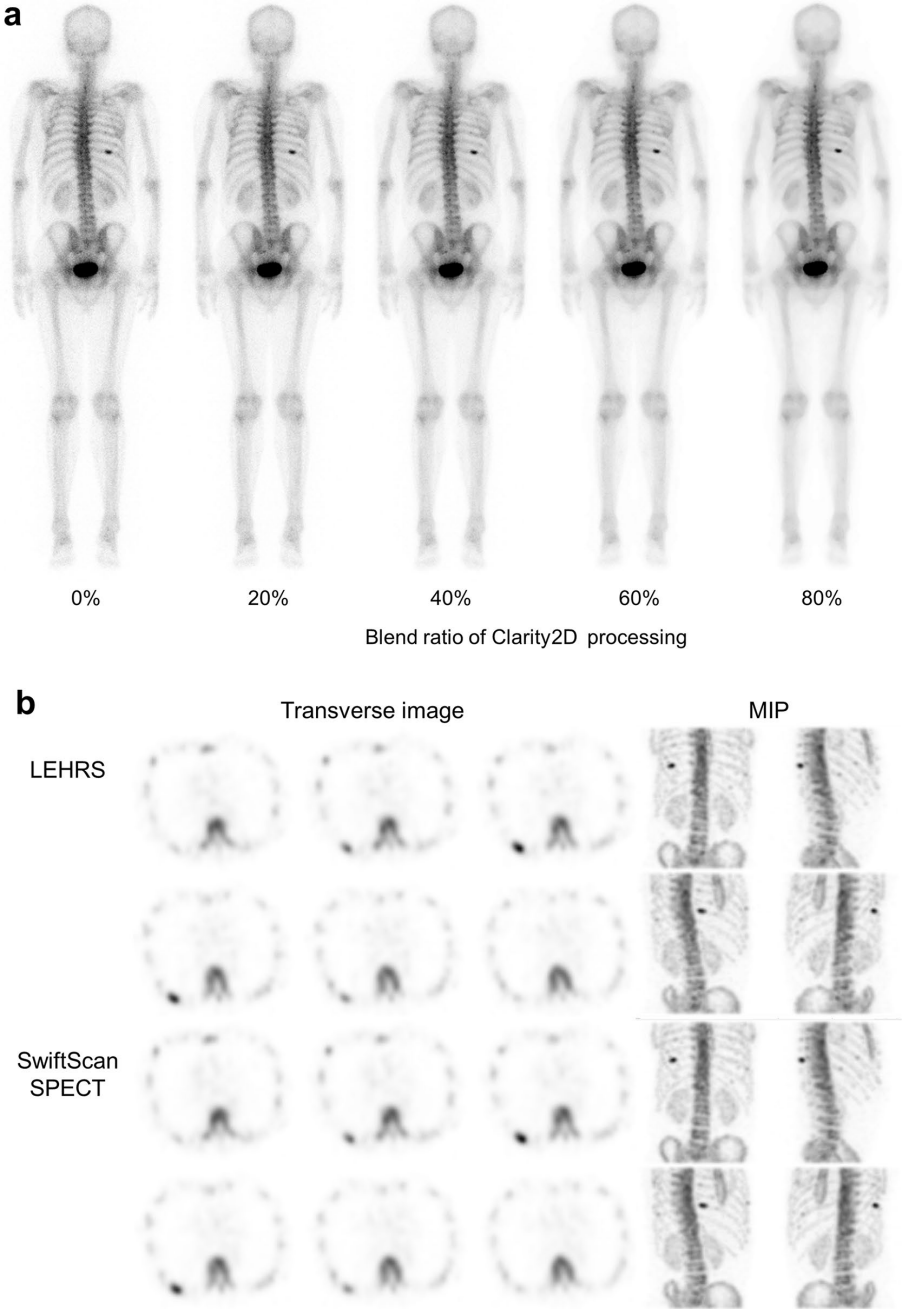
Clinical image of SwiftScan planar and SPECT images. (**a**) Shows a whole-body (WB) image with and without SwiftScan planar processing. The blend ratio of Clarity 2D processing 0% means the WB image without SwiftScan planar processing. (**b**) Shows transverse and maximum intensity projection (MIP) images with and without SwiftScan SPECT. The first and second rows show the images of low-energy high-resolution and sensitivity (LEHRS) collimator without SwiftScan SPECT. The third and fourth rows show the SwiftScan SPECT images.

The whole-body images with SwiftScan planar processing of different blend ratios and Swift Scan SPECT in the clinical cases were shown in Fig. 6. The CNR between abnormal uptake of right 9th rib and background region for the original image with LEHRS collimator was 14.4, and the CNRs of SwiftScan image with a blend ratio of 20%, 40%, 60% and 80% were 18.4, 23.0, 31.8 and 42.1, respectively. The CNR of the SwiftScan planar image for clinical images showed a higher value with an increase in a blend ratio of Clarity 2D processing as well as phantom study. The CNRs of conventional SPECT with LEHRS collimator and SwiftScan SPECT were 157 and 317, respectively. SwiftScan SPECT image showed a higher CNR compared with conventional SPECT.

## Discussion

The number of bone SPECT imaging has increased with the spread of a SPECT/CT scanner, thereby total examination time for the bone scan has been extended^21^. Therefore, shorter examination time is important to improve the patient burden. It has been reported in the previous study that the SwiftScan system using the LEHRS collimator has a higher sensitivity than the conventional scan with the LEHR collimator^10^. Furthermore, the Clarity 2D processing for the SwiftScan planar image can reduce the image noise while maintaining at least an equivalent contrast recovery and spatial resolution, and the image quality of the SwiftScan planar can be adjusted by the blend ratio of original and the processed images after noise reduction and contrast enhance. However, the blend ratio of Clarity 2D processing was only evaluated using the constant value in the previous study. Furthermore, the phantom study was evaluated using the National Electrical Manufacturers Association (NEMA) body phantom filled with radioactive water^22^; however, this phantom was unable to reproduce bone structure, attenuation and scatter radiation. Therefore, we evaluated the image quality of the SwiftScan planar image at different blend ratios using a more flexible bone phantom filled with a mixed solution of bone equivalent material and ^99m^Tc.

Although planar images with LEHR and LEHRS collimator have similar image quality, the planar image with LEHRS collimator showed a slightly lower %CV than that with LEHR collimator owing to higher pixel counts for improvement of sensitivity. SwiftScan planar image has a higher CNR and constant contrast ratio with an increasing blend ratio to maintain image contrast and remove the noise of Clarity 2D processing^6–9^. However, short-time acquisition of less than 50% could not sufficiently assure the image quality. In particular, the image uniformity of SwiftScan planar image less than 50% acquisition time could not be improved using Clarity 2D processing. On the other hand, the acquisition time was more than 75%, SwiftScan planar images with a blend ratio of Clarity 2D processing more than 40% have good image quality. Therefore, the SwiftScan planar image was found to require a count of 75% acquisition time or higher and blend ratio of Clarity 2D processing more than 40% to maintain the image quality.

The contrast ratio decreased with smaller tumor size due to the partial volume effect^23^. Furthermore, the increase of CNR with the increase in Clarity 2D processing was greater for larger tumor sizes, and the reduction in %CV was also similar. Differences in the effects of CNR and %CV on different tumor sizes were caused by the synergistic effects of partial volume effect and Clarity 2D processing with noise reduction. Although Clarity 2D processing is performed with contrast enhancement processing by the Lucy-Richardson algorithm with the empirical kernel, the SwiftScan planar image was influenced by the partial volume effect as well as conventional image^8,9^. The partial volume effect markedly reduces quantitative accuracy^24^. Therefore, we should validate the partial volume effect correction to improve the quantitative accuracy in the future.

Some technologies similar to the SwiftScan planar effect have been already reported. The Planar processing software using the Pixon method can improve the image quality of whole-body bone scintigraphy by the effect of a noise-reducing post-processing algorithm that minimizes image noise in the processed image while preserving vital information from the original data^25–27^. However, the Planar processing software using the Pixon method does not apply to SPECT images as well as SwiftScan planar technology. On the other hand, the nonlinear diffusion (NLD) processing can also reduce statistical noise while preserving the edge signal information^28^. Furthermore, the NLD processing can be applied to SPECT images^29^. If the SwiftScan planar technology is extended to SPECT images, the image quality of SPECT will be even better.

The projection data of the SwiftScan SPECT image added the acquisition counts owing to data acquisition during detector movement. Therefore, added acquisition counts resulted in a relative decrease in statistical noise, so that the SwiftScan SPECT image can obtain good image quality as well as a previous study^10^.

We evaluated the SwiftScan planar and SPECT systems under the routine acquisition and image reconstruction parameters. It is necessary to demonstrate how SwiftScan planar and SPECT images are affected by the acquisition and image reconstruction and processing conditions. In particular, SwiftScan SPECT changes the counts obtained during the detector movement depending on the setting of the sampling angle. Therefore, we have to demonstrate the effects of sampling angles. Although we evaluated bone scintigraphy as a target, SwiftScan planar and SPECT techniques can be potentially applied to other scintigraphy, for which further technological development is expected.

## Conclusion

SwiftScan planar and SPECT images were able to reduce the image noise compared with planar and SPECT images with a LEHR collimator, which resulted in good image quality with a high CNR. The SwiftScan planar image was able to reduce the acquisition time by 25% when the blend ratio of Clarity 2D processing set to 40% or more.
